# On Anœmic Murmurs

**Published:** 1846-10

**Authors:** H. M. Hughes

**Affiliations:** Assistant Physician to Guy’s Hospital


17. *On Anœmic Murmurs.* By H. M. Hughes, M. D., Assistant Physician to Guy's Hospital.* In certain states of the system, or it may be, with certain conditions of the circulating fluid, as in chlorosis, or in anæmia from hemorrhage, or from other causes, murmurs frequently arise from the passage of the blood, independently of absolute disease of the heart or great vessels. These are termed *anæmic murmurs* or "*chlorotic bruits.*"

They are ordinarily of the softer kind, and resemble the blowing of a pair of bellows ("*bruit de soufflet*"), but they are sometimes quite harsh, and resemble the rougher morbid sounds, as that of filing or sawing ("*bruit de râpe* and *bruit de scié*").

They are very generally supposed to be confined to the aortic openings. This is certainly a mistake. They are most assuredly very frequently connected with the pulmonary artery, in which murmurs, quite independent of any disease of the vessel or of its valves, are far from uncommon.

Murmurs often arise from some body pressing upon this vessel; as a solid mass, the result of pleurisy, of pneumonia, or of phthisis, or enlarged bronchial glands, abscesses of the anterior mediastinum, &c., &c. The murmurs frequently also coexist with chlorosis, or with other forms of anæmia. Are these latter murmurs, then, whether in the pulmonary artery or in any other part of the circulating system, to be distinguished with tolerable certainty from morbid sounds, the result of organic obstruction within or without the heart or large vessels?

Generally speaking, they may, I believe, be distinguished from each other; but they certainly cannot always be so; and never with absolute certainty by the mere character of the murmur alone. There are, I feel assured, some examples of these anæmic murmurs, which can be proved to be simply functional, and not to arise from organic disease of the heart or its vessels, or from pressure upon them, only by the results of treatment.

Let, then, the student be careful not to assert too confidently that a patient on the one hand, has organic disease of the heart, or great vessels, merely because he has a harsh murmur over the aorta, an occasionally irregular rhythm, and a vibrating pulse, which usually coexist with an anæmic condition of the body, or he may cause unnecessary alarm and

* Clinical Introduction to the Practice of Auscultation, &c., p. 221.

anxiety; nor let him, upon the other hand, too hastily determine, that, because a murmur is soft, and his patient is an hysterical girl, with a pale face, and is subject to leucorrhœa and to amenorrhæa, that she has no organic disease; or some day, to his great surprise, grief, and mortification, and possibly also to his disgrace, he may find she has died suddenly with diseased heart.

Anæmic murmurs, however, it may be stated, are very local and are generally pretty much confined to the situation of the sigmoid valves, either aortic or pulmonary, or both; they do not follow the course of the large vessels so fully, or so frequently, as do the murmurs arising from disease of the valves, or of the arteries: they occur only during the systole of the ventricles; and as they cannot arise from regurgitation through the mitral valve, they are not heard very distinctly below the left nipple; they are always, so far as I know, accompanied with a smart smacking impulse; they generally disappear for a time while the individual is quiet, mentally as well as bodily, if by that quiet the heart assumes a natural impulse; and they are always diminished, and generally disappear entirely, under suitable treatment.

The origin of the anæmic mumurs has latterly been very generally attributed to a watery condition, or a diminution of ordinary viscidity, of the blood: in consequence of which it is believed that the particles of the fluid move more easily over each other, are therefore more freely agitated, and thus give rise to the vibrations which produce the murmur. This may have some, and perhaps an important, influence in producing them.

But there are other circumstances which also appear to play an important part in their causation. The principal of these is the remarkably quick and sudden contraction of the ventricles; in consequence of which the fluid contents of the cavities are propelled through the comparatively small area of the mouths of the large arteries in a shorter time than during the leisurely contractions of health, or the frequent, but not sudden, contractions existing in some other forms of disease. Though, therefore, no actual contraction exists, an obstruction is practically produced by the increased velocity with which the blood is propelled through the aortic and pulmonary openings. The increased agitation in the fluid thence arising it is at least probable, has a principal part in the production of anæmic murmurs.

If the heart beat quietly, and the impulse be natural, however decided the pallor of the face, and whatever the watery condition of the blood, no murmur, I believe, exists, when no mechanical obstruction is present.

It is also possible that the *quantity* of the circulating fluid is decreased in such cases, in addition to its *quality* being altered, and that while, by the elasticity of their coats, the arteries are capable of accommodating themselves to the diminished quantity of the fluid, the cavities of the ventricles retain their normal capacity, and that on this account an absolute, as well as a comparative obstruction, may exist to the transit of the blood.

Concurrently with these anæmic murmurs at the origin of the large arteries, there is often heard, upon the application of the stethoscope to the side of the neck, a curious sort of humming noise, which ceases when firm pressure is exerted upon the jugular vein at a point above that on which the end of the stethoscope is placed. It is continuous, not intermittent like the arterial murmur, and is, therefore, sometimes called the "continous humming," as well as the "venous murmur," —"*bruit de diable*," &c.

It most probably depends upon partial obstruction to the quickened flow of blood through the veins. Strong pressure causes it to cease; but without pressure, *directly or indirectly applied*, it is, I believe, never heard. Like the anæmic murmur of the arteries, it is supposed to be associated with a watery condition of the blood, and it is, we are told, a frequent, if not a constant, attendant upon that state of the system with which such a watery condition of the blood is a concomitant. This statement is not made from my own observation, but if true, the venous hum may perhaps be considered a useful assistant indication of the anæmic state.

But great obstruction to the blood may, as has been previously hinted, exist; extensive disease may be present in the valves of the heart, or in the large arteries, and yet no murmur may be heard. This arises from circumstances which may be, as they have already been partially, illustrated by the stream, in which a certain rapidity of the current is necessary to produce such an agitation of the water as will give rise to sound. Though the bottom of a rivulet be very uneven and its banks exceedingly irregular, yet if the current be not tolerably strong, little or no riple will be produced, and no sound will be generated. It is just so with the blood; rapidity of the current of the blood, as well as obstruction thereto, is necessary to produce such an agitation among the particles of the fluid as will give rise to sound.

Hence it often happens that a heart with extensive disease of the valves may be without murmur while the patient is quiet, and the circulation is slow; though immediately the circulation is accelerated, either by physical exertion or by mental emotion, a murmur becomes distinct. Hence, also, it happens, that when the cavities of the heart become greatly distended, in consequence either of the magnitude of the obstruction, or of defective nervous power, the ventricles are frequently incapable of acting upon and propelling their contents with sufficient force to produce a murmur. The channel is irregular enough, but the rapidity of the current, and of the resulting vibrations, is not equal to the generation of sound. Hence, likewise, it arises, that when fluid is present to a large amount in the pericardium, the heart may be so oppressed with the accumulation upon the exterior, that, though great obstruction exist within, no murmur is produced. Thus it will often be observed that when the obstruction is greatest, the murmur, if even it be heard at all, is very feeble; and that when the obstruction is small, the murmur is very loud; thus also, in persons who, for weeks and months, and even years, have presented notably morbid cardiac sounds, these sounds, if the individuals are not carried off suddenly, very frequently, or perhaps even generally, cease altogether some days before death.

The cause of this, as before stated, is either that the heart does not contract with sufficient power, or if it act forcibly, that it cannot act upon, and propel through the contracted orifices, the large quantity of blood which distends its cavities with a rapidity sufficient to give rise to sound.

Let, then, the student ever bear in mind the truth, that mere obstruction is not in itself sufficient, but that a certain force or rapidity of the circulation must be necessarily combined with that obstruction, to give rise to morbid endocardial sounds. Murmurs may exist without any obstruction of an organic kind; but without a certain degree of force in the circulating current they cannot exist.—*Southern Med. and Surg. Journal.*

